# Ultrasonic Sensing and Actuation in Laminate Structures Using Bondline-Embedded d35 Piezoelectric Sensors

**DOI:** 10.3390/s18113885

**Published:** 2018-11-11

**Authors:** Hussain Altammar, Anoop Dhingra, Nathan Salowitz

**Affiliations:** Mechanical Engineering Department, University of Wisconsin, Milwaukee, WI 53211, USA; Dhingra@UWM.edu (A.D.); Salowitz@UWM.edu (N.S.)

**Keywords:** shear-mode piezoelectric transducers, flexural waves, laminate structures, structural health monitoring

## Abstract

Ultrasonic systems employing embedded piezoelectric transducers have seen increased interest in recent years. The ability to sense, actuate, and analyze the wave propagation modes in engineering structures has been fundamental to the advancement of ultrasonic structural health monitoring (SHM). This paper presents a study into the sensing and actuation properties of shear-mode (d35) piezoelectric transducers made of lead zirconate titanate (PZT) that are internally embedded in the bondline of laminate structures. The manuscript presents analytical analysis, finite element simulation, and experimental validation building from an individual piezoelectric element to a full laminate structure. The validated model was then used to perform a parametric study into the effects of d35 PZT transducer size on the strength of actuation and sensing output signal. The selectivity of d35 PZT sensors was also investigated by generating multiple wave modes in the laminate structure and inspecting the output signals. The d35 PZT sensors were found to selectively detect only certain modes of the wave propagation providing a fundamental hardware filter that could be employed to simplify signal analysis and processing. The results of this study indicate that d35 PZTs embedded in the bondline have multiple properties that can potentially be employed for ultrasonic SHM.

## 1. Introduction

This paper presents an investigation into the properties of ultrasonic waves actuated and sensed by shear-mode piezoelectric transducers embedded within the bondlines of laminate structures. Structural sensing based on ultrasonic waveform analysis, employing embedded piezoelectric actuation and sensing elements, has greatly advanced in recent years. Analysis of ultrasonic wave propagation is one of the most advanced techniques for damage detection systems employing embedded sensors, known as structural health monitoring (SHM). Significant research has sought to preferentially actuate, sense, and analyze specific symmetric and antisymmetric wave propagation modes to detect specific damage forms. Past studies have found that damage in adhesive bonds interacts the most with waveforms that produce shear strain in the bondline, making preferential actuation and sensing useful [1,2]. The majority of this work has employed thin piezoelectric transducers polarized and electrically actuated in the same direction (i.e., the *x*_3_-direction) and adhered to the outer surface of the structure to function as actuators and sensors. The application of an electric field in the polarized direction of the piezoelectric transducer actuates normal (d33) and perpendicular (d31) strains in the piezoelectric material, which generate elastic waves in the structure. The wave propagation actuated and sensed by surface-mounted PZT transducers have been studied by many researchers [3,4,5,6,7,8,9]. Another class of PZT piezoelectric transducers that are polarized along their length (i.e., in *x*_1_-direction) induce shear strain in the piezoelectric material when an electric field is applied in the thickness direction. The shear deformation has also been found to have a stronger coupling coefficient (d35) than d33 or d31, indicating shear mode PZTs have stronger electromechanical coupling for sensing and actuation [10]. For example, PZT-5A has piezoelectric coupling coefficients of d31 = −171 pm/V, d33 = 374 pm/V, and d35 = 584 pm/V. These transducers are known as shear-mode (d35) PZT piezoelectric transducers. There have been relatively few studies considering the use of d35 PZT piezoelectric transducers for sensing and actuation of ultrasonic waves [11,12,13,14]. Kamal and Giurgiutiu [11,12] investigated shear horizontal (SH) waves induced by shear-mode piezoelectric transducers mounted on the surface of aluminum plates. A closed-form solution was derived to predict the wave propagation of SH waves in a simple structure. They also studied the electromechanical (EM) impedance of shear-mode PZTs attached to the surface of a plate using multiphysics finite element (FE) simulations with experimental validation. Similarly, Zhou et al. [13] presented the use of a shear-mode PMNT piezoelectric wafer (crystal) mounted on the surface of an aluminum plate to generate SH waves for damage detection of surface defects. They conducted FE simulations for sensing and actuation of SH waves and validated the results experimentally.

Some other studies involved using shear-mode PZTs as actuator mechanisms [15,16,17]. Benjeddou and Deu [15,16] presented a 3-D analytical formulation of transverse shear actuation and sensing to study the static response and the resonant frequencies of a piezoelectric laminated plate with a d35 PZT layer sandwiched between two graphite/epoxy composite plates. They found that variation in the PZT thickness does not significantly affect the in-plane displacements or transverse deflection of the laminate plate thickness. The optimum position of PZT transducers was found to be the mid-plane of the laminate plate. Furthermore, a 3-D FE simulation for static analysis of a sandwiched beam with a d35 PZT embedded as the central layer was presented by Koutsawa et al. [17]. Displacement, stress, and electric potential fields of beams were investigated using 3D FEM analyses. It was found the mechanics of 3D shear actuated beams with a full PZT layer are very complex. In another study, the shear response of piezoceramic transducers was analyzed to investigate the piezoelectric and dielectric nonlinearity of soft and hard piezoelectric materials at 100 Hz for position control applications [18]. Baillargeon and Vel [19] investigated the effectiveness of d35 PZT actuators for active damping of a cantilever sandwich beam. Benjeddou et al. [20] have conducted static and modal analyses based on theoretical and numerical methods in order to compare the performance of shear actuation and extension actuation mechanisms in cantilever beams by recording beam tip deflection. The existing literature indicates that shear-mode piezoelectric actuators have found more extensive use in the field of active vibration control than in the field of nondestructive evaluation. This paper presents an investigation into the properties and benefits of shear-mode PZT piezoelectric transducers in sensing and actuation ultrasonic waves while being internally embedded within the bondline of laminate structures.

Investigations into ultrasonic inspection of bonded joints have consistently found that waves that create shear stress and strain in the joint are the most sensitive to bondline damage. Nagy [1] investigated the use of a conventional C-scan configuration to generate compression and shear waves at the interface of double-layer specimens. It was observed that shear waves have higher sensitivity to joint defects than compression waves. Kundu et al. [2] also conducted harmonic ultrasonic testing on glass specimens using two transducers in a pitch-catch arrangement. They found the first antisymmetric Lamb wave mode (A_0_) was sensitive to the presence of joint defects.

Bondline-embedded sensors have been investigated by many researchers using different types of sensors, including piezoelectrics. Blanas et al. [21] have embedded bimorph piezo-composite film in composite plates for detection of acoustic emission signals. Zhuang et al. [22] and Dugnani et al. [23] investigated embedding piezoelectric sensors into the bondline of laminate structures to inspect the adhesive bond integrity by monitoring their electromechanical (EM) impedance. Because EM impedance methods rely on structural stiffness to predict the bondline integrity, they are limited to the region occupied by piezoelectric sensors.

Despite a relatively large body of work in bondline-embedded sensors and wave propagation analyses, most of the work was concerned with either conventional d31 piezoelectric wafer active sensors (PWAS), d33 PZT interdigitated transducers (IDT), or surface-mounted d35 PZT transducers (SH-PWAS). This paper presents a study into the properties of ultrasonic waves of bondline-embedded d35 PZT transducers in laminate structures from the individual transducer to a full a laminate structure employing analytical methods, finite element analysis, and experimental validations. The analysis of the piezoelectric response of a free-d35 PZT element along with its resonant mode shapes was conducted to understand the nature of shear-mode piezoelectric transducers. Experimental analysis of a double-layered laminate specimen with two d35 PZT transducers embedded within the bondline in a pitch-catch configuration was performed next. The propagating waves in the laminate specimen were identified to have the characteristics of flexural (antisymmetric) waves and these findings were further validated using a multiphysics FE simulation. The voltage signals obtained from the laminate specimen at different actuation frequencies were then compared with the FE voltage signals to determine the frequency bandwidth of the d35 actuator. Based on the validated model, a parametric study was performed by varying the thickness and length of d35 PZT transducers while monitoring the actuation strength and sensor voltage signal. The size of the bondline-embedded d35 PZT transducers was found to have a significant influence on the actuation strength and sensing ability of the transducers. The selectivity of d35 PZT sensors was investigated by generating multiple wave modes in the laminate structure with comparison to a simulated surface-mounted d31 PZT sensor output signal. The paper concludes with the main findings and observations in this study.

## 2. Shear Mode of Free-d35 PZT

**Figure 1:**
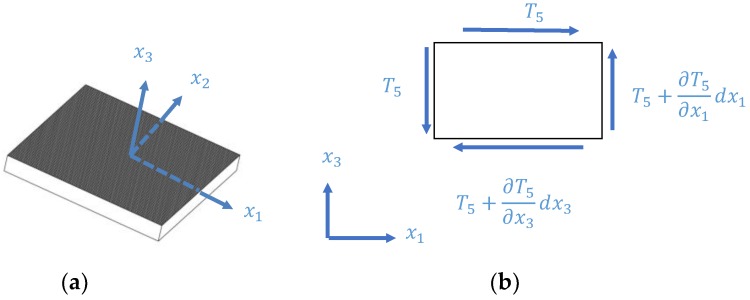
(**a**) Schematic of a shear-mode plate piezoelectric transducer polarized in the $x_{1}$-direction; (**b**) infinitesimal shear element.

The modal analysis of a free shear-mode PZT element yields the natural mode shapes and frequencies that are essential to understand the electromechanical behavior of the PZT element when embedded in a structure. Identifying the mode shapes and natural frequencies supports selection of actuation frequencies as some systems intentionally excite and avoid resonances. Knowledge of the mode shapes can also help to predict the propagating waves in the structure. This analysis begins with an analytical derivation that was performed based on an established theory, followed by finite element analysis and experimental validation. The idealized geometry of a piezoelectric element is presented in Figure 1a, which is polarized in the $x_{1}$-direction excited by an electric voltage applied in the $x_{3}$-direction, on the top and bottom electrodes, causing the transducer to oscillate periodically in accordance with the frequency of the induced electric field. The transducer has a length of *l* in the $x_{1}$-direction, a width of *b* in the $x_{2}$-direction, and a thickness of *h* in the $x_{3}$-direction.

### 2.1. Analytical Approach

Piezoelectric materials are modeled as having a linear relation between the mechanical and the electrical properties. The general constitutive equations in tensorial form are expressed in IEEE standard format [24].

(1) $$T_{ij}=c_{ijkl}^{E}S_{kl}-e_{kij}E_{k}$$

(2) $$D_{i}=e_{ikl}S_{kl}+\varepsilon_{ik}^{T}E_{k}$$

In Equations (1) and (2), $T_{ij}$ is the mechanical stress, $c_{ijkl}^{E}$ is the material stiffness coefficient at the zero electrical field, $S_{kl}$ is the mechanical strain, $E_{k}$ is the electrical field, $D_{i}$ is the electrical displacement, $\varepsilon_{ik}^{T}$ is the material dielectric permittivity at zero mechanical stress, and $e_{kij}$ and $e_{ikl}$ are the piezoelectric stress coupling coefficients between the mechanical and electrical variables. For a piezoelectric transducer polarized in the $x_{1}$-direction with an electric field induced in the $x_{3}$-direction, the transducer is assumed to have a predominant shear motion in the $x_{1}-x_{3}$ plane and decoupled shear strains among the principal planes. The shear strain is assumed to be uniform along the transducer length and width. Also, the transducer was assumed to have transversely isotropic material properties and its volume remains substantially constant in shear vibration. The general constitutive equations for a piezoelectric transducer having a linear relation between the mechanical and the electrical properties can be expressed in IEEE standard format [24] as:

(3) $$T_{5}=c_{55}^{E}S_{5}-e_{35}E_{3}$$

(4) $$D_{3}=e_{35}S_{5}+\varepsilon_{33}^{T}E_{3}$$

In Equations (3) and (4), $T_{5}$ is the shear stress in the $x_{1}-x_{3}$ plane, $S_{5}$ is the mechanical shear strain in the $x_{1}-x_{3}$ plane, $c_{55}^{E}$ is the stiffness coefficient, $E_{3}$ is the electric field, $D_{3}$ is the electric displacement, $\varepsilon_{33}^{T}$ is the material dielectric permittivity at zero mechanical stress, and $e_{35}$ is the stress coupling constant between the electric field applied in the $x_{3}$-direction and mechanical shear strain in the $x_{1}-x_{3}$ plane. The impedance response of a free shear-mode piezoelectric transducer was derived by Kamal et al. [11]:

(5) $$Z=\frac{\hat{V}}{\hat{I}}=\frac{1}{i\omega C}\left[\frac{k_{35}^{2}}{k_{35}^{2}+1}\frac{\tan(\beta )}{\beta }-1\right]$$

In Equation (5), $\hat{V}$ is the magnitude of the electric voltage, $\hat{I}$ is the magnitude of the electric current, the electric capacitance is $C=\varepsilon_{33}^{T}A/h$, *A* is the surface area of the transducer, the shear electromechanical coupling factor is $k_{35}^{2}=e_{35}^{2}/\varepsilon_{33}^{T}c_{55}$, β=γh/2, the wavenumber is $\gamma =\omega /c_{s}$, ω is the angular frequency, and the shear wave speed in the material is $c_{s}=\sqrt{{\bar{c}}_{55}/\rho }$, and ${\bar{c}}_{55}=c_{55}^{E}+e_{35}^{2}/\varepsilon_{33}^{T}$. The impedance response can show the EM resonances and anti-resonances while varying the frequency of the applied voltage. The impedance response given Equation (5) is slightly different from reference [11], because it accounts for the additional stiffness from the electric effect. More details on the formulation of the piezoelectric response can be found in reference [25].

### 2.2. FE Simulation

The finite element (FE) method was also used to analyze the mode shapes and natural frequencies of a free piezoelectric transducer. A square plate with the dimensions of 15 mm × 15 mm × 1 mm was modeled, matching experimental specimens. A multiphysics harmonic analysis was conducted in ANSYS 17.0 to simulate a 3-D piezoelectric transducer using a brick coupled-field element, SOLID226. Harmonic analysis was conducted with an alternating electric voltage applied on the surface electrodes to excite the transducer at a frequency sweep ranging from 10 kHz to 7 MHz. The structural and electromechanical material properties of a piezoelectric transducer polarized in the $x_{1}$-direction given in IEEE standard format [25] were used to define the elasticity matrix, the piezoelectric stress coupling constants, and the permittivity matrix as shown in Equation (6). 

(6) $$\begin{array}{l} \left[c\right]=\left[\begin{array}{cccccc} 110.9 & 75.1 & 75.1 & 0 & 0 & 0\\ 75.1 & 120.4 & 75.2 & 0 & 0 & 0\\ 75.1 & 75.2 & 120.4 & 0 & 0 & 0\\ 0 & 0 & 0 & 22.6 & 0 & 0\\ 0 & 0 & 0 & 0 & 21.1 & 0\\ 0 & 0 & 0 & 0 & 0 & 21.1 \end{array}\right]GPa\\ \left[e\right]=\left[\begin{array}{ccc} 15.784 & 0 & 0\\ -5.351 & 0 & 0\\ -5.351 & 0 & 0\\ 0 & 0 & 0\\ 0 & 0 & 12.295\\ 0 & 12.295 & 0 \end{array}\right]\frac{C}{m^{2}}\;\;\;\left[\varepsilon^{T}\right]=\varepsilon_{o}\left[\begin{array}{ccc} 1581 & 0 & 0\\ 0 & 1851 & 0\\ 0 & 0 & 1851 \end{array}\right] \end{array}$$

In Equation (6), $\varepsilon_{o}$ is the vacuum permittivity and has a value of 8.854 μF/m. A convergence analysis was carried out to ensure the appropriate number of divisions by monitoring the third EM frequency.

### 2.3. Experiment

Ten shear-mode piezoelectric elements with dimensions of 15 mm × 15 mm × 1 mm were tested to obtain their EM resonances. These elements were made of APC850 material (equivalent to Navy II) and manufactured by APC International, Ltd. (Mackeyville, PA, USA) [26]. A test fixture was constructed composed of a couple of springs that functioned as a c-clamp, allowing adjustment of the pressure on the specimen and the jaw gap. Specimens were placed between the end-tips of two rods that gently touched the specimen under test. A 100 Ω resistor was placed in series with the specimen and connected to a KEYSIGHT 33500B Series waveform generator (Santa Rosa, CA, USA) at the other terminal. A MDO3014 Mixed Domain Oscilloscope manufactured by Tektronix (Beaverton, OR, USA) was used to obtain voltage measurements across the specimen and the entire circuit. The impedance of shear-mode PZTs was calculated by the following expression:

(7) $$Z=R_{s}\frac{V_{i}}{V_{o}}$$

In Equation (7), Z is the electrical impedance, $R_{s}$ is a constant resistance, $V_{i}$ is the voltage applied to the circuit, and $V_{o}$ is the voltage across the sensing resistor and the PZT element. The tone burst signal in a frequency sweep mode transmitted to the specimen was generated by the waveform generator varying continuously from 10 kHz to 7 MHz.

**Figure 2:**
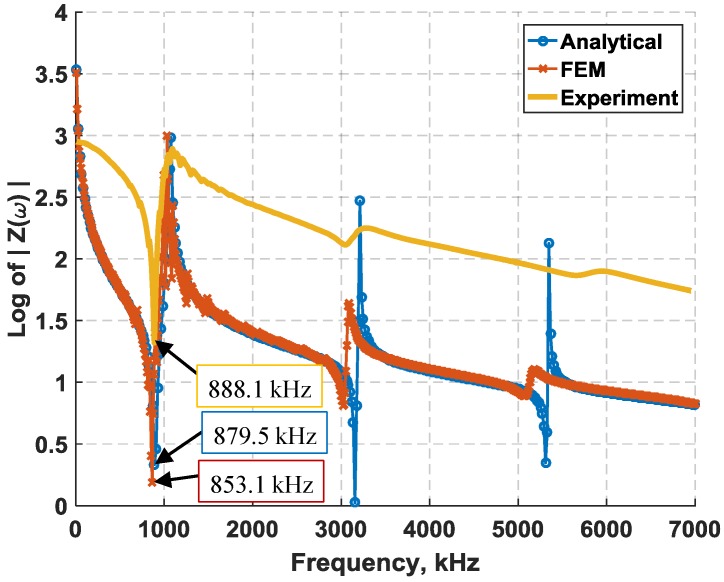
Piezoelectric response of a shear-mode piezoelectric transducer obtained using the analytical approach, finite element, and experiment.

The EM resonant frequencies of shear-mode transducers found using experimental, analytical, and FE approaches for the first three resonances match relatively well as shown in Table 1. The experimental EM resonances are the average values of 10 PZT elements. The close match between the analytical and FE results indicate a possible mismatch between the idealized model properties and the actual test specimens, which could include the transducer thickness, structural dimensions, or electromechanical properties, as these were found to be the primary parameters affecting the shear-mode piezoelectric response. The standard thickness tolerance of a shear-mode element is also ±0.05 mm. Therefore, a different thickness within the tolerance limits can significantly shift the locations of electromechanical resonances in the analytical and FE analyses closer to the actual resonances. Overall, the response of the experimental, FE, and analytical approach in Figure 2 are comparable.

**Figure 3:**
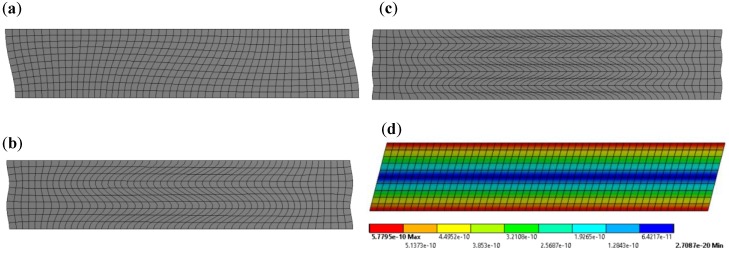
Finite element mode shapes of free d35 PZT element: (**a**) First mode at 853 kHz; (**b**) second mode at 3001 kHz; (**c**) third mode shape at 5051 kHz; (**d**) total deformation (mm) at 30 kHz.

The frequencies of electromechanical resonances and anti-resonances can also be identified from the piezoelectric response. The first three mode shapes obtained from the FE model are displayed in Figure 3. The mode shape of a free-d35 PZT element actuated at 30 kHz is also shown in Figure 3. It can be noted from the natural mode shapes that the d35 PZT element undergoes almost negligible displacement at the neutral axis with sinusoidal behavior across the thickness. Additionally, the higher modes show limited displacement indicating it may not be advantageous to actuate at these frequencies. On the other hand, the actuation of d35 PZT at 30 kHz induces linear displacement across the thickness, with maximum displacement in the top surface and minimum displacement in the bottom surface. This analysis will be further discussed in the wave propagation analysis in the next section.

## 3. Actuation and Sensing of d35 PZTs

Experimental and numerical analyses were performed to study the actuation and sensing properties of d35 PZT transducers embedded within the bondline of laminate structures to actuate and sense ultrasonic waves. The test geometry used in both simulations and experiments consisted of two d35 PZTs sandwiched between two 1 mm thick aluminum sheets, which were bonded together with a layer of adhesive of Hysol EA 9394 epoxy (Bay Point, CA, USA). The aluminum sheets were machined to a 305 mm × 15 mm × 1 mm in size. The d35 PZTs were placed 130 mm apart with their polling direction aligned along the length. This layout was determined to avoid overlapping signals from reflection. The waveform signals obtained from the laminate specimen were compared with the FE voltage signals. Simulation was also used to perform a parametric study on the effects of varying the actuation frequency, and the length and thickness of d35 PZT transducers.

### 3.1. Experimental Approach

**Figure 4:**
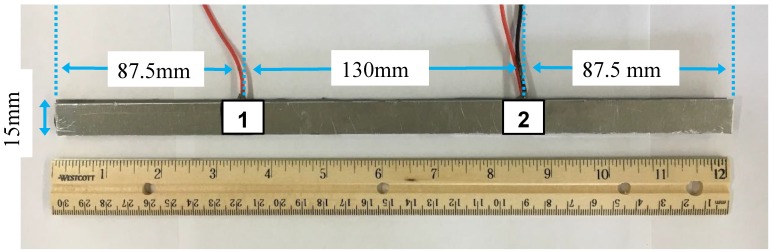
Fully prepared sample with two square d35 PZTs embedded within the bondline with their polling direction along the length of the aluminum sheet.

In this experiment, the laminate specimen was prepared as previously stated, consisting of two d35 PZTs sandwiched between two 6061-T6 aluminum sheets that were bonded together with a layer of adhesive. The aluminum sheets were machined to a 305 mm × 15 mm × 1 mm in size. The shear-mode d35 PZTs were adhered to one aluminum sheet that would serve as a common ground using Chemtronics CircuitWorks CW2400 conductive epoxy (Kennesaw, GA, USA) [27]. The d35 PZTs were placed 130 mm apart with their polling direction aligned along the aluminum length. The same conductive epoxy was used to attach thin wires to the individual hot terminals of the PZTs. A fully prepared specimen consisting of the aluminum sheet with d35 PZTs and wiring are shown in Figure 4. Hysol EA 9394 was then used to bond the aluminum sheets together [28]. This epoxy also served as an insulator, protecting the hot terminals of the PZTs from shorting against the second aluminum plate. The adhesive thickness was controlled by placing 1 mm thick spacers on the short edges (vertical boundaries) and applying pressure on the specimen while curing. The adhesive layer thickness was measured after curing at 1 ± 0.2 mm. Both d35 PZTs were made of APC 850 piezoelectric ceramic material with properties given in [25,26]. 

**Figure 5:**
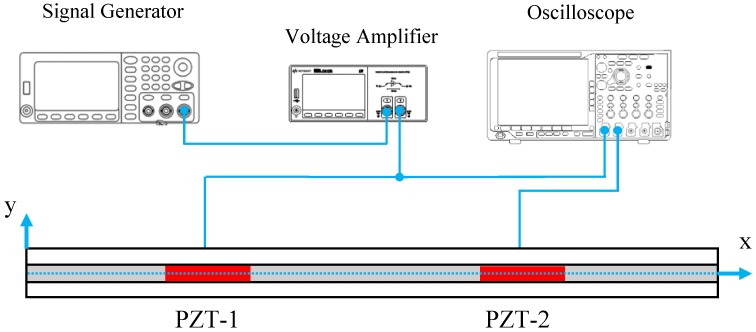
Schematic of the experimental setup for testing laminate specimen consists of two d35 PZT embedded within the bondline in a pitch-catch configuration.

The experimental setup implemented to test the laminate specimen is shown in Figure 5. The d35 PZT transducer labeled as PZT-1 was connected to a KEYSIGHT 33500B Series waveform generator [29] and the output signal was amplified using a Krohn-Hite 7602M Wideband Amplifier (Brockton, MA, USA) [30]. Also, both d35 PZTs were connected to a Tektronix MDO3014 Mixed Domain Oscilloscope [31] to simultaneously record voltage signals across the actuator and the sensors. The specimen was tested in a pitch-catch configuration by actuating one PZT with a 5-peak Hanning windowed signal at different center frequencies.

### 3.2. Numerical Approach

Simulation of a laminate structure with internally embedded d35 PZTs provided insights into their actuation and sensing properties along with the wave propagation modes. A 2D numerical simulation was implemented to analyze the actuation and sensing properties of d35 PZT transducers embedded in the bondline of laminate structures. The piezoelectric properties of the transducers were simulated using multiphysics analyses that couple electric and mechanical fields simultaneously in the solution process. The numerical simulation was performed in ANSYS 17.0. The overall geometry of the laminate structure is the same as the laminate specimen shown in Figure 4 for comparison purposes. The laminate structure consisted of two 6061-T6 aluminum sheets bonded together with Hysol EA9394 adhesive. Two 15 mm square d35 PZTs with 1 mm thickness were modeled in the bondline in a pitch-catch configuration to actuate and sense the propagating elastic waves in the structure. The material properties of the laminate structure components, including aluminum, adhesive, and PZT, are provided in Table 2. The electromechanical behavior of the d35 PZTs was simulated using a coupled-field element, PLANE223. To accurately simulate the wave propagation, it is required to have a mesh size of at least 1/10 of the wavelength [32]. Therefore, the element size of the couple-field elements and structural elements were set as 0.1 mm. The aluminum and the adhesive were modeled with a structural element, PLANE183. PZT-1 was simulated as an actuator and PZT-2 as a sensor through the application of electrical boundary conditions. A 5-peak Hanning windowed tone burst signal with a magnitude of 190 Volt peak-to-peak was applied to PZT-1. The interface regions in the laminate structure were defined as fully bonded joints using contact and target elements. The simulation results, including waveform signals, displacement, and stress distributions along the thickness of the laminate structure, were obtained and are discussed in the subsequent section.

### 3.3. Wave Propagation Analysis

**Figure 6:**
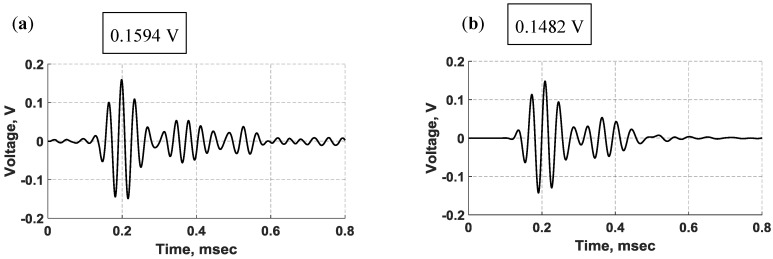
Waveform signals sensed by d35 PZT transducers embedded in the bondline of the laminate structure: (**a**) Experiment; (**b**) simulation.

Based on the configuration of the shear-mode PZT transducers in the bondline of the laminate structure and their mode shapes discussed in Figure 3, the shear actuation was expected to produce transverse shear stress, which is coupled to bending deformation through the classic beam theory, actuating flexural (antisymmetric) waves propagating in the structure [33]. Unless otherwise specified; throughout this analysis, a 5-peak Hanning windowed tone burst signal at 30 kHz was applied to the actuator. The waveform signals produced by the bondline-embedded d35 PZT sensors from experiment and simulation are provided in Figure 6. There are two main wave packets in the received signals. The first arrival traveled 130 mm directly from the actuator to the sensor followed by its reflection from the far boundary. Close inspection of experimental signals revealed minor electromagnetic crosstalk. The crosstalk was found to match the actuation signal in time, shape, and frequency, confirming its transmission to the data acquisition system through electromagnetic phenomena and not strain wave propagation. The first wave packet in the FE voltage signal is slightly damped as compared to the experimental signal. However, the secondary reflections in the FE signal are relatively over-damped when compared with the experimental signal. This may be due to the numerical damping factor that was imposed on the model to ensure the solver stability and convergence to the correct solution. The elastic waves had to travel 130 mm and the time of flight (TOF) for the first arrival wave packet from the actuator to the sensor was measured to be 119.2 µs and 110.1 µs for the simulation and experiment, respectively. Furthermore, the experimental signal also reached the sensor slightly faster than the FE signal with a time difference of about 9.1 µs, resulting in a 1089.5 m/s group velocity for the FE first arrival and 1180.7 m/s group velocity for the experimental first arrival, respectively. This analysis combined with the structural deformation analysis that follows confirms that flexural waves were actuated and propagated in the structure by bondline-embedded d35 PZTs. 

**Figure 7:**
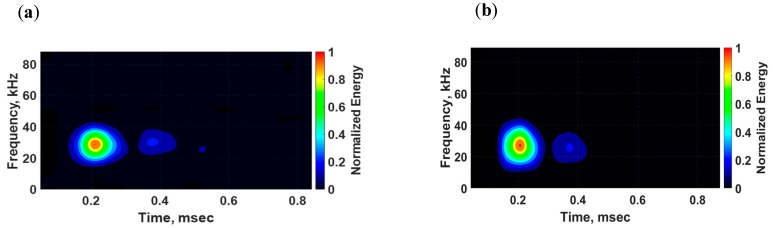
Time-frequency spectrums of voltage signals sensed by d35 PZT transducers embedded in the bondline of the laminate structure: (**a**) Experiment; and (**b**) simulation.

The group velocity of flexural waves is twice the phase velocity of individual waves within the flexural wave packet, making flexural waves highly dispersive [34]. Dispersive effects on wave propagation are dependent on the frequencies of both the carrier signal and the modulation window, resulting in distortions of both the shape and magnitude of the propagating signal. To qualitatively examine the dispersion effect in this analysis, the time-frequency spectrums of sensed signals were determined and are displayed in Figure 7. The dispersion effect is slightly noticeable in the spectrums due to the short distance between the PZT transducers. By closely inspecting the spectrums, it can be observed that the dispersion effect is more noticeable on the second wave packet than on the first wave packet because the reflection wave packet traveled a longer distance than the first major arrival wave packet.

**Figure 8:**
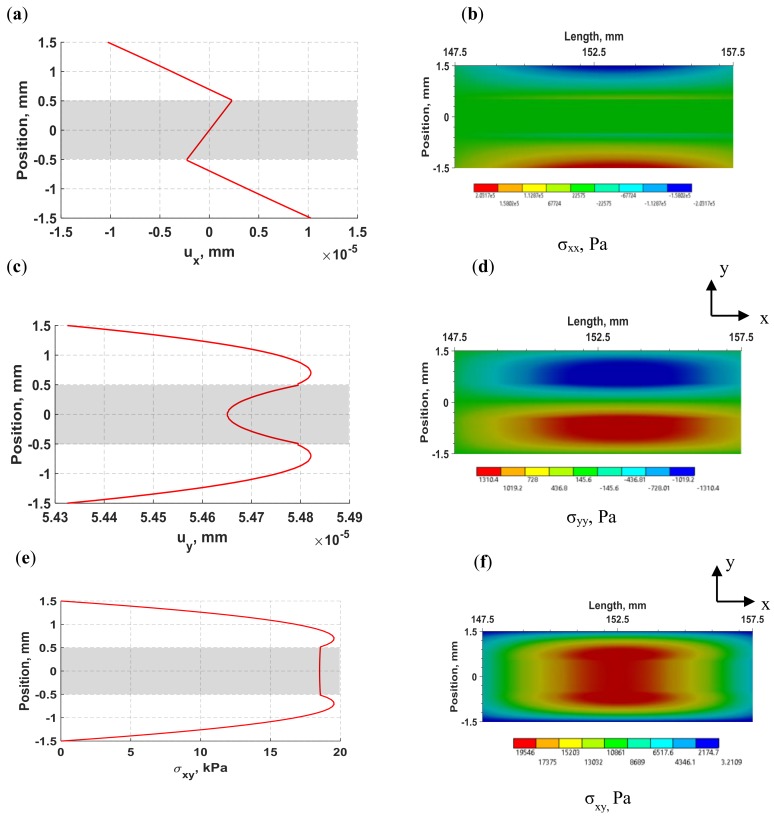
1D through-thickness distributions (left) and 2D full-field view (right) of flexural waves in laminate structure induced by a d35 PZT actuator with a 30 kHz 5-peak signal. (**a**) Axial (*x*) displacement; (**b**) stress in the *x*-direction; (**c**) lateral (*y*) displacement; (**d**) stress in the *y*-direction; (**e**) and (**f**) in-plane shear stress.

The distributions of normal displacements and stresses of flexural waves in the laminate structure induced by the d35 PZT actuator at 30 kHz are displayed in Figure 8. The distributions were obtained at 138.3 µs. The 1D through-thickness distributions were calculated for a set of nodes located at the middle of the structure (152.5 mm) and the shaded regions represent the position of the adhesive layer with respect to the upper and lower substrates. The 2D full-field views for stress components were calculated for a section of the laminate structure located between 147.5 mm and 157.5 mm. In Figure 8a,b, the normal displacement in the *x*-direction shows linear displacement over each layer with zero displacement at the neutral axis, resulting in maximum tension in the upper plate and maximum compression in the lower plate. The distribution of normal displacement in the *y*-direction (*y*) shown in Figure 8c is maximum in the substrates and minimum in the adhesive layer, but due to negligible difference between the extreme values of the lateral (*y*) displacement, it can be considered uniform through the thickness over the period of wave propagation. It may be noted from Figure 8a through to Figure 8d that the magnitude of lateral (*y*) displacement is about five times larger than the axial (*x*) displacement, but the normal lateral (*y*) stress is negligible compared to the axial (*x*) normal stress. The distribution of shear stress in Figure 8e,f shows an increasing shear stress from the outer surface to the interface region in the aluminum substrates while the adhesive layer maintains a constant and comparatively high shear stress.

The propagating waves exhibit the characteristics of flexural waves, which induce high lateral (*y*) displacement coupled with maximum axial (*x*) stress in the outer surface and maximum transverse shear stress near the neutral axis of the structure. In summary, a good agreement between experimental and FE voltage signals was achieved, supporting the effectiveness of d35 PZT transducers to actuate and sense flexural waves while being embedded in the bondline of laminate structures as well as validating the FE modeling procedure for the subsequent analyses throughout this study.

### 3.4. Frequency vs. Sensing Voltage Relation

There are several factors that can influence the behavior of actuated flexural waves in laminate structures, such as actuation frequency, structural stiffness, and the geometry of the PZT transducer, including its thickness and area. A parametric study was performed investigating the relation between the actuation frequency and the senor output voltage using the validated model. The actuation voltage was kept constant throughout this study and structural deformation was inspected in the model. This was performed by keeping the geometry constant and varying the frequency using simulation and experiments.

**Figure 9:**
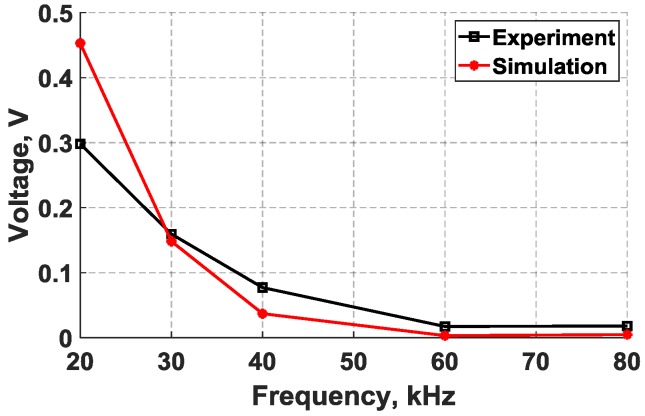
Maximum sensor voltage amplitude versus actuation frequency for flexural waves sensed and actuated by bondline-embedded d35 PZT transducers using a 5-peak actuation signal with a constant actuation voltage.

The results for the maximum voltage in the sensed signals from the simulation and experiment are plotted in Figure 9 for comparison. The experimental results were obtained from the same specimen presented in Figure 4. Likewise, the simulation results were based on a 2-D FE model that was performed by following the modeling procedure discussed in Section 3.2. It can be noted from Figure 9 that as the actuation frequency increases, the amplitude of the voltage decreases, resulting in weaker voltage signals. The flexural mode was found to be strongly attenuated above approximately 40 kHz in the received signals. This frequency range is considered relatively small and that can be attributed to the structural stiffness constraining the movement of the d35 PZT actuator as well as the size and shape of the actuator. Structural damping can potentially attenuate the signal, but the distance between the transducers is considered too short to cause significant reduction in the magnitude of voltage signals.

**Figure 10:**
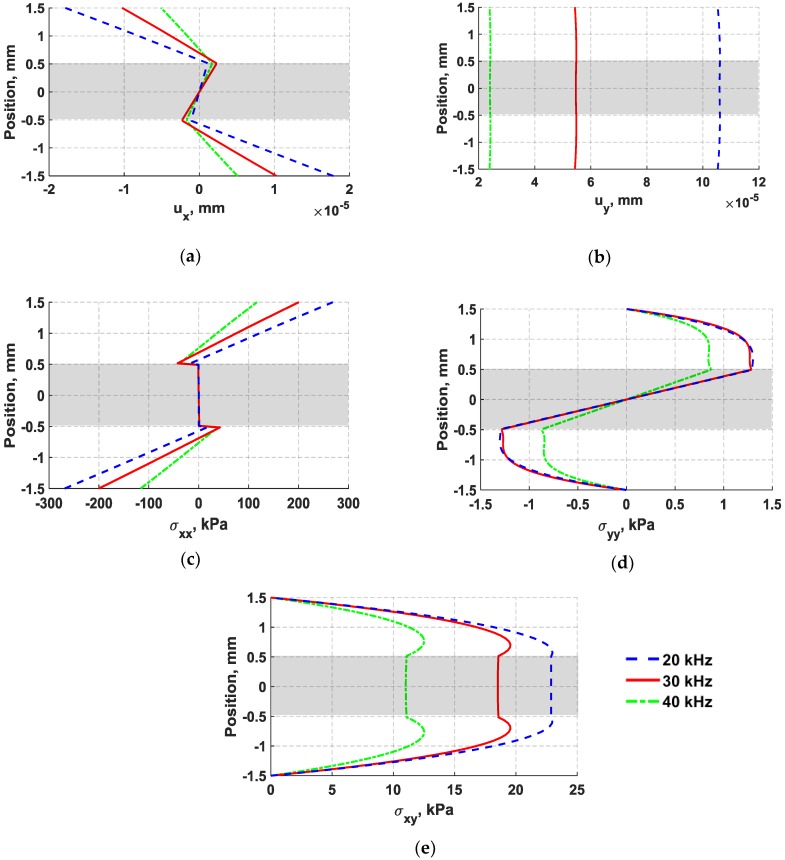
Comparison of through thickness displacements and in-plane stresses of flexural waves induced by bondline-embedded d35 actuated with 5-peak Hanning window signal at three actuation frequencies: 20 kHz (blue dashed line), 30 kHz (red solid line), and 40 kHz (green dash-dotted line). (**a**) axial (*x*) displacement; (**b**) lateral (*y*) displacement; (**c**) stress in *x*-direction; (**d**) stress in *y*-direction; (**e**) in-plane shear stress.

In Figure 10, the distributions of through thickness displacements and in-plane stresses obtained from nodes located at the middle of the structure (152.5 mm) are plotted for three different frequencies, including 20 kHz, 30 kHz, and 40 kHz. The shaded region in Figure 10 indicates the location of the adhesive layer with respect to the aluminum substrates. In Figure 10, it can be seen that an increase in the actuation frequency results in a magnitude reduction of all normal displacement and stress components. The voltage reduction observed from the d35 sensor signal is expected to be predominantly caused by reduction in the in-plane shear stress, specifically across the thickness of the adhesive layer. The decrease in frequency caused the shear stress distribution to have the maximum shear stress concentrated within the adhesive layer. As previously mentioned in the literature, waveforms that generate shear strain have been found to be very effective for detecting joint defects by several researchers [1,2,35]. Therefore, it is important to note that the high level of change of the shear stress in the bondline as compared with other existing stresses in the laminate structure provides an opportunity to design a d35 transducer such that the strength of the in-plane shear stress is focused along the thickness of the adhesive layer for monitoring the integrity of bondlines.

### 3.5. Parametric Study of d35 PZT Size

A numerical parametric study varying the thickness and length of d35 PZT transducers was conducted to investigate the effects on their sensing and actuation while internally embedded in the bondline of laminate structures. The geometry of the laminate structures along with the configuration of d35 PZTs considered herein is the same as the laminate specimen shown in Figure 4. The thickness of the laminate structure was kept the same in order to maintain a constant structural stiffness throughout the analyses. Similar to what was previously presented, a 5-peak Hanning windowed tone burst signal with a center frequency of 30 kHz was used in the analysis. However, the geometry of both transducers was modified simultaneously for each simulation.

**Figure 11:**
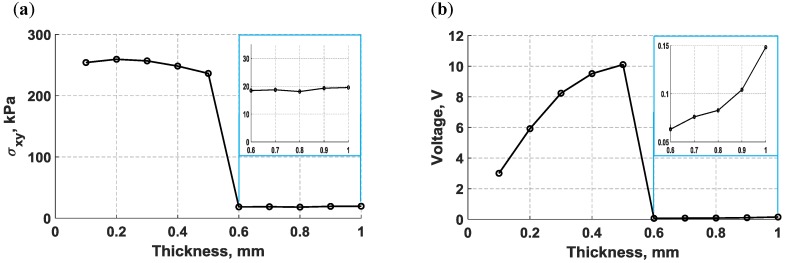
A numerical parametric study varying the thickness of bondline embedded d35 PZT transducers and actuating with a 5-peak tone burst signal at 30 kHz; (**a**) maximum in-plane shear-stress generated by the d35 PZT actuator, and (**b**) maximum voltage produced by the d35 PZT sensor.

To analyze the thickness effects on the sensing and actuation of bondline-embedded d35 PZTs, the maximum in-plane shear stress generated by the actuator and the maximum voltage produced by the sensor were plotted against the PZT thickness in Figure 11a,b respectively, as the thickness was varied from 0.1 mm to 1.0 mm in an increment of 0.1 mm. Inspection of Figure 11a reveals that the actuation strength of d35 PZT shows complex behavior. Small thicknesses show the strongest actuation, which diminishes as the thickness increases until a thickness of 0.5 mm is reached. At this point, there is an abrupt drop in actuation strength with minimal variation as the thickness continues to increase. In Figure 11b, an opposite behavior was observed from the d35 PZT sensor for the same thickness, indicating that thicker d35 PZT sensors can produce stronger output voltage signals than thinner d35 PZTs. The low voltage amplitudes at a thickness greater than 0.5 mm in Figure 11b can be attributed to the low actuation strength of thick d35 PZT actuators.

**Figure 12:**
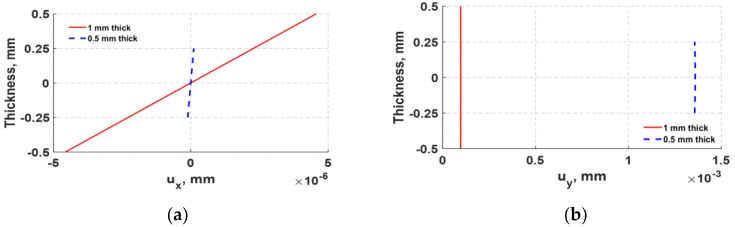
Comparison of through-PZT thickness displacements for a 1 mm actuator (red solid line) and 0.5 mm actuator (blue dashed line) excited with a five-peak tone burst signal at 30 kHz. (**a**) Axial (*x*) displacement; (**b**) lateral (*y*) displacement.

**Figure 13:**
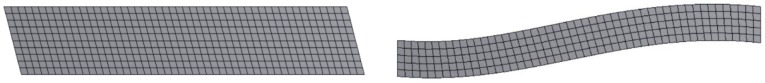
Total deformation of bondline-embedded d35 PZT actuators with thicknesses of 1 mm (**left**) and 0.5 mm (**right**) excited with a five-peak tone burst signal at 30 kHz.

The abrupt shift in actuation shown in Figure 11a was further examined by analyzing the structural deformation of 1 mm and 0.5 mm d35 PZT actuators. The normal displacements displayed in Figure 12 were obtained from a set of nodes located at the left vertical edge of both d35 PZT actuators. It can be observed from Figure 12 that the 1 mm d35 PZT actuator generates higher axial (*x*) displacement, but lower lateral (*y*) displacement than the 0.5 mm d35 PZT actuator, indicating a radical change in the response of the bondline-embedded d35 PZT actuator when its thickness was reduced below 0.5 mm. Furthermore, the total deformation of the actuators embedded in the bondline of the laminate structure are displayed in Figure 13. The results indicate that the 1 mm d35 PZT primarily exhibits shear mode actuation to produce flexural waves while 0.5 mm d35 PZT shows flexural mode actuation. This general behavior is believed to be a combined effect of the change in electric field intensity in the actuator as the thickness changes along with the stiffness ratio of the actuator relative to the structure. The results indicate that the stiffness ratio is the main factor for the transition in the behavior of the bondline-embedded PZT transducer at 0.5 mm. It should be noted from Figure 11 that there is an optimal stiffness ratio for which d35 PZT actuators generate strong flexural waves in a laminate structure. Further investigation is necessary as outlined in the future work section.

**Figure 14:**
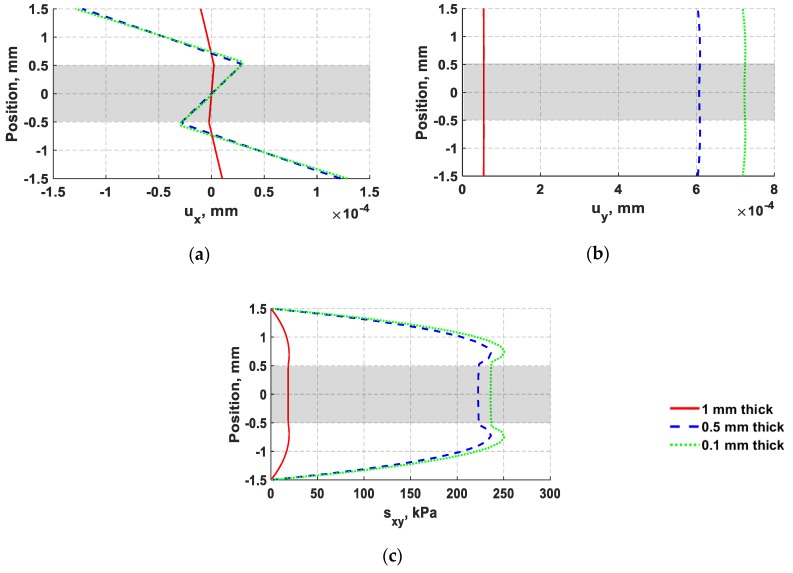
Comparison of through thickness displacements and in-plane stresses of flexural waves at 30 kHz with varying the thickness of d35 PZT transducers as follows: 1 mm (red solid line), 0.5 mm (blue dashed line), and 0.1 mm (green dotted line). (**a**) Axial (*x*) displacement; (**b**) lateral (*y*) displacement; (**c**) in-plane shear stress.

The distributions of through thickness normal displacements and in-plane shear stress for 0.1 mm, 0.5 mm, and 1 mm actuators are also provided in Figure 14. The effect of varying PZT thickness on each normal displacement component is not linear, showing higher change on lateral (*y*) displacement than axial (*x*) displacement. However, the lateral (*y*) displacement of flexural waves is expected to have little influence on d35 PZT sensors, especially when the propagating waves have a long wavelength of 39 mm as in this case. A d35 PZT sensor is fundamentally efficient in sensing in-plane shear stress because these waves align well with its natural vibration mode as shown in Figure 3. Therefore, d35 PZT sensors are not expected to be capable of sensing in-plane symmetric axial (*x*) displacement. This concept will be further examined in the next section.

**Figure 15:**
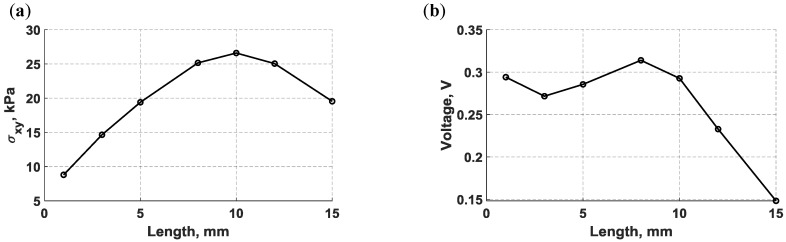
A numerical parametric study varying the length of bondline embedded d35 PZT transducers and actuating a 5-peak tone burst signal at 30 kHz; (**a**) maximum in-plane shear-stress generated by the d35 PZT actuator, and (**b**) maximum voltage produced by the d35 PZT sensor.

The length of bondline-embedded d35 PZT transducers was also varied from 1 mm to 15 mm. The maximum shear stress, which represents the actuation strength herein, was monitored while varying the PZT length and is plotted in Figure 15. As can be noted from Figure 15a, the actuation strength increases and reaches its maximum level at 10 mm, and then decreases as the actuator is approaching 15 mm in length. To analyze the sensing performance, the maximum voltage produced by d35 PZT sensors were recorded and plotted against their length in Figure 15b. d35 PZT sensors have higher sensitivity for the propagating waves when shorter d35 PZT sensors were used as this can be discerned from Figure 15b.

In summary, the numerical parametric study shows that designing bondline-embedded d35 PZT transducers imposes differing preferences for actuator and sensor geometries. Therefore, finding the optimum pair of d35 PZT transducers can maximize the signal to noise ratio and the wave propagation distances, which are essential in building a robust structural health monitoring system.

## 4. Selectivity of d35 PZTs

**Figure 16:**
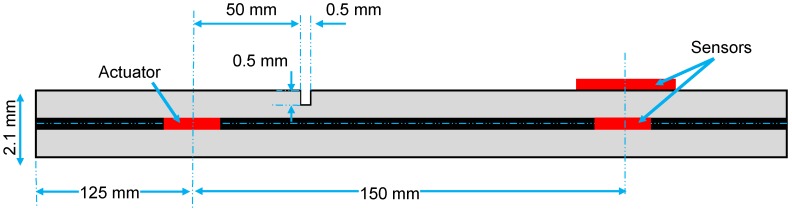
Schematic of a laminate structure with two d35 PZT transducers (3 mm × 0.1 mm) embedded in the bondline and a d31 PZT sensor (6 mm × 0.25 mm) mounted on the surface. A notch (0.5 mm × 0.5 mm) is introduced in the top aluminum layer.

When ultrasonic waves propagate through a structural defect, the interaction of waves and the defect often cause wave scattering and mode conversion. Therefore, a laminate structure with a surface notch was simulated using FE multiphysics analysis to demonstrate the selectivity of bondline-embedded d35 PZT sensors to different modes propagating simultaneously in a structure. In Figure 16, a laminate specimen with two d35 PZT transducers (3 mm × 0.1 mm) internally embedded in the bondline and a d31 PZT sensor (6 mm × 0.25 mm) mounted on the surface are shown. A square surface notch of a 0.5 mm width and depth was introduced at 50 mm from the actuator. The distance between the transducers and the location of the notch was determined such that the scattered modes would not overlap for demonstration purposes of the d35 sensor’s selectivity. The d31 PZT was integrated in the model to highlight the selectivity characteristics of the d35 PZT sensor by comparing the sensed waveform signals obtained from both the d35 PZT and d31 PZT sensors. The material properties of the laminate structure are given in Table 2. A 5-peak Hanning windowed actuation signal at 250 kHz was applied to the actuator to generate flexural (antisymmetric) waves in the laminate structure.

**Figure 17:**
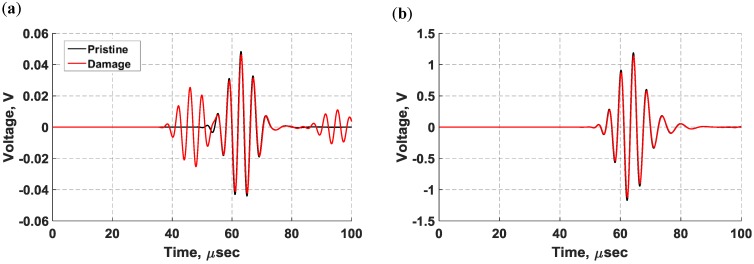
Sensor signals from the pristine state (black) and damaged state (red) at 250 kHz for: (**a**) d31 PZT sensor mounted on the surface of the laminate structure; (**b**) d35 PZT sensor embedded within the bondline.

**Figure 18:**
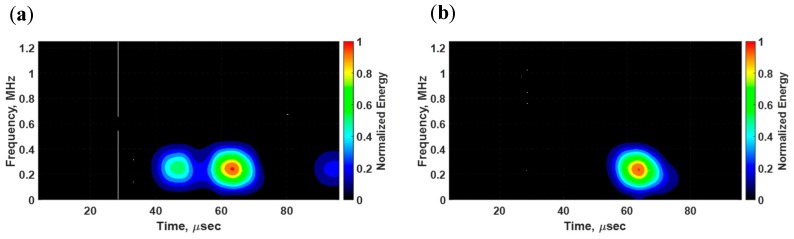
Time-frequency spectrums of voltage signals obtained from a (**a**) d31 PZT sensor and (**b**) d35 PZT sensor for a laminate structure with a notch excited with a d35 PZT actuator at 250 kHz.

In Figure 17, the voltage signals from the pristine state (black) and damaged state (red) are superimposed for comparison purposes. The propagating waves were collected without a notch, identifying this state as the pristine state, and the damaged state was defined with the notch being inflicted on the surface. As can be quickly noticed, the signals from the surface-mounted d31 PZT sensor exhibit multiple modes propagating in the structure whereas the signals from the bondline-embedded d35 sensor only show sensitivity to the antisymmetric signal that was present in both the pristine and damaged states. At a low actuation frequency of 250 kHz, the symmetric modes traveled faster than the antisymmetric modes [36]. In the d31 sensor signal, two extra wave packets were generated due to the presence of the notch as shown in Figure 17a. The first wave packet did not appear in the signal from the pristine state and was identified as a symmetric mode that resulted from the mode conversion that occurred at the notch. The next wave packet was very similar to the pristine signal and was an antisymmetric mode. The third wave packet was the reflection of the symmetric mode from the boundary. The time-frequency spectrum for the damaged state in Figure 18a shows that all wave packets had about the same center frequency. The results of the d35 PZT sensor in Figure 17b and Figure 18b show a complete absence of the symmetric modes in the voltage signals and time-frequency spectrum. This observation suggests that d35 PZT sensors have strong selectivity to sense antisymmetric waves while rejecting symmetric waves.

As previously shown in Figure 8, the d35 PZT sensor is mainly sensitive to in-plane shear stress that are found to be negligible on the surface of the structure. As a result, the interaction between shear and the notch is expected to be small as shown in Figure 17b. The damage effect in the d35 signal is relatively small and manifested in the form of a phase shift and magnitude reduction. On the contrary, normal displacements shown in Figure 8 are found to be significant on the surface of the structure. This makes surface-mounted d31 (and d33) PZT sensors sensitive to any distortion in the profile of normal displacements, indicating the location of the d31 sensor on the surface as very effective. Based on this observation, joint defects are also expected to interact with stronger in-plane shear in the bondline, yielding more accurate information about the integrity of the bondline than surface mounted PZTs.

In summary, the results suggest that d35 PZT sensors offer a valuable merit by largely capturing antisymmetric wave modes in the media. Multimodal superimposed waves of differing modes can coexist in the plate, resulting in complex patterns that make interpreting the data to characterize defects a challenging task [37,38]. The selectivity of d35 sensors give them a useful advantage in signal processing because it makes the interpretation of the data more efficient and the outcomes more reliable. This advantage also enables a simple comparison between reflected and transmitted waves from the pristine state and damaged state. Filtering symmetric modes in received signals significantly reduced the complexity of signal processing and this could potentially enhance the process of SHM as well.

## 5. Conclusions

This paper presented an investigation into the properties of ultrasonic waves actuated and sensed by d35 PZT piezoelectric transducers internally embedded within the bondline of laminate structures. The modal analysis of a free-d35 PZT transducer was conducted using analytical, numerical, and experimental approaches and the results were found to match well. A double-layered aluminum specimen with two d35 PZT transducers embedded within the bondline in a pitch-catch configuration was prepared and tested. A multiphysics FE simulation of the laminate structure with two bondline-embedded d35 PZT transducers was also conducted to support the analysis of the propagating waves in the laminate specimen. The group velocities of experimental and FE voltage signals as well as the distributions of normal displacements and stresses induced by the propagating waves showed that the elastic waves generated by the d35 PZT actuator exhibit the characteristics of flexural waves coupled with strong transverse shear stress across the thickness of the adhesive layer. The voltage signals obtained from the experiment and simulation were found to be in a good agreement, supporting the effectiveness of 35 PZT transducers to actuate and sense flexural waves while being embedded in the bondline of laminate structures.

The frequency versus sensing voltage relation was investigated by varying the actuation frequency and monitoring the amplitude of the received signals using FE simulations and then validating the results experimentally. The results from experiment and simulation indicated that there is an inverse relation between the actuation frequency and the maximum voltage. It was also noted that the strength of the shear stress can be concentrated across the thickness of the adhesive layer at lower actuation frequencies. As previously noted in the literature, waves that place the bondlines in shear either through transvers shear coupled with antisymmetric waves or shear waves were found to be the most effective to detect joint defects. Therefore, this provides an opportunity to design d35 PZT actuators such that they generate strong in-plane shear strains largely aimed to target the bondline region for adhesive joint evaluation.

A parametric study was performed where the thickness and length of d35 PZT transducers were varied while monitoring the actuation strength and the sensed voltage signal. The size of bondline-embedded d35 PZT transducers was found to have a significant influence on the actuation strength and the sensing ability of d35 PZT transducers. It was found that thicker and shorter d35 PZT sensors can produce stronger signals compared to thinner and longer d35 PZT sensors. On the contrary, d35 PZT actuators were noticed to exhibit the opposite response to d35 PZT sensors with more complex behavior when the thickness and length were varied. This demonstrated that the design of d35 PZT sensors and actuators to be embedded within a bondline for ultrasonic inspection favor differing geometries. This created an optimization challenge if a given transducer is intended to function as both an actuator and a sensor, generating strong actuation without compromising sensing ability. 

The selectivity of d35 PZT sensors was also investigated in simulation by comparing voltage signals obtained from a bondline-embedded d35 PZT sensor and surface-mounted d31 PZT sensor. Mode conversion from wave interaction with a surface notch provided a collection of symmetric and antisymmetric waves in the laminate structure. It was found that d35 PZT sensors offer a selective hardware filter that primarily captures antisymmetric wave modes in the laminate structure while suppressing symmetric wave modes. It is known that complex superposition of wave modes often results in difficulties in damage detection and signal analysis. The selectivity of d35 PZT sensors could be employed in signal processing to makes the interpretation of the data more efficient, reducing uncertainty, and leading to more reliable analysis outcomes.

## 6. Future Work and Vision

Future work will include experimentally validating the parametric study of the d35 PZT size while being embedded in the bondline of laminate structures. Furthermore, the selectivity of d35 PZT sensors to antisymmetric wave modes and rejecting symmetric modes needs to be experimentally verified through comparison of voltage signals from surface-mounted d31 and bondline-embedded d35 PZT sensors. Experimental comparison with d31 PZT signals will both confirm the selectivity of the d35 PZT and provide insight into the relative efficiency of the technique presented herein compared to traditional surface mounted d31 techniques for actuating and sensing ultrasonic waves. A comprehensive study of the efficiencies of actuating and sensing different types of ultrasonic waves using the various electromechanical coupling properties of PZTs in different structural locations would also be insightful, but is expected to be complicated by the fundamentally different deformations. Because this work is expected to be of importance for the field of SHM of adhesive joints, testing the ability to use flexural waves generated and sensed by bondline-embedded d35 PZT transducers to detect different joint defects, including cracks, delaminations, kissing bonds, and disbands, should be performed as well. Placing PZT transducers in the bondline also provides protection for the transducers from environmental, chemical, and physical hazards. Miniaturization of d35 PZT transducers and supporting systems also needs to be further investigated.

## Figures and Tables

**Table 1:** Results summary of electromechanical resonances for a shear-mode APC-850 piezoelectric element: Experiment, closed-form, and FE.

Natural Mode	Experiment (MHz)	Closed-Form (MHz)	Finite Element (MHz)
1	0.888	0.879	0.853
2	3.139	3.028	3.001
3	5.637	5.092	5.051

**Table 2:** Material properties of the shear-mode [25,26], Hysol EA9394 [28], and Aluminum 6061.

Property	Unit	Symbol	PZT-5A	Adhesive	Aluminum
Young’s Modulus	10^9^ N/m^2^	$Y_{11}$	61.0	4.24	68.9
	10^9^ N/m^2^	$Y_{33}$	53.2	4.24	68.9
Shear’s Modulus	10^9^ N/m^2^	$G_{12}$	22.6	1.46	25.9
	10^9^ N/m^2^	$G_{13}$	10.5	1.46	25.9
Poisson’s ratio	1	$\nu_{12}$	0.35	0.45	0.33
	1	$\nu_{13}$	0.44	0.45	0.33
Density	kg/m^3^	ρ	7600	1360	2700
Dielectric permittivity	8.854 µF/m	$\varepsilon_{11}$	1851	------	------
	8.854 µF/m	$\varepsilon_{33}$	1581	------	------
Piezoelectric coefficient	10^−12^ m/V	$d_{15}$	584	------	------
	10^−12^ m/V	$d_{31}$	−171	------	------
	10^−12^ m/V	$d_{33}$	374	------	------
